# Comparison of attempts and plans to quit tobacco products among single, dual, and triple users

**DOI:** 10.18332/tid/169663

**Published:** 2023-09-13

**Authors:** Jieun Hwang

**Affiliations:** 1 Department of Health Administration, College of Health and Welfare, Dankook University, Cheonan-si, Republic of Korea

**Keywords:** tobacco products, cigarette smoking, electronic nicotine delivery systems, heated tobacco products, vaping

## Abstract

**INTRODUCTION:**

Tobacco users are categorized as single, dual, and triple users based on the number of tobacco products (cigarettes, e-cigarettes, and heated tobacco products) used. This study addressed a literature gap by examining how adult Korean tobacco users’ quit attempts/plans differed based on the user type, and the associated psychosocial and subjective health-related factors.

**METHODS:**

We used a questionnaire to examine participants' self-reported health, stress, health concerns, health behavior, tobacco addiction, intentions/plans to quit, and demographic characteristics. Data were analyzed using chi-squared tests, one-way analysis of variance, and multiple linear regression.

**RESULTS:**

Of the 1288 tobacco users, 55.4%, 28.3%, and 16.4% were single, dual, and triple users, respectively. Self-rated health and stress were lowest among single users and highest among triple users. Most user types had intentions/plans to quit, especially triple users. Quit attempts and plans increased with increasing health behaviors and time elapsed before first tobacco use in the morning, but decreased with higher stress and self-rated addiction.

**CONCLUSIONS:**

Intentions/plans to quit tobacco use varied based on the type of tobacco user. Multiple users had higher self-rated health, plans to quit, and self-reported addiction; they considered themselves healthy or engaged in healthy behaviors to offset problems from tobacco use and used multiple tobacco products to quit smoking. Highly stressed users had fewer plans to quit and used tobacco for stress relief. Thus, the provision of accurate information about tobacco products and stress management is important to promote successful quitting.

## INTRODUCTION

Cigarettes continue to dominate the global tobacco market. As of 2018, cigarettes accounted for 91% of the total market value of tobacco products, while electronic nicotine delivery systems (ENDS, e-cigarettes) and heated tobacco products (HTPs) combined account for 2.2%^1^. However, the problem is that the market for ENDS and HTP has been experiencing steady growth. Between 2014 and 2019, the HTP market alone saw explosive growth, with global sales increasing from 100000 devices and 15.3 million HTP sticks in 2014 (valued at $5.6 million) to 12.8 million devices and approximately 69.5 billion sticks sold in 2019 (valued at $15.2 billion)^2^. According to Euromonitor International, the global e-cigarette market also experienced significant growth, with its value more than tripling from $6.8 billion in 2014 to $20.2 billion in 2019^2^.

The diversification of the tobacco market has led to the categorization of tobacco users into single, dual, and triple users based on the types of tobacco products (cigarette, e-cigarette and/or HTP) used. In 2018, a survey of 7000 adults in South Korea found that among single users, 14.4% used cigarettes, 0.9% used e-cigarettes, and 1.3% used HTPs^3^. Among dual users, 2.0% used cigarettes + e-cigarettes, 5.0% used cigarettes + HTPs, and 0.5% used e-cigarettes + HTPs^3^. Triple users accounted for 3.4%^3^. In 2020, a study of 4689 e-cigarette users aged ≥15 years in Japan found that 50.9% were single users, 35.2% were dual users, and 13.8% were triple users^4^. In short, e-cigarette or HTP users are more likely to be dual or triple users, implying that they have a higher level of nicotine dependence than single users, requiring greater effort to quit smoking^3-5^.

Interests or concerns about health are the strongest motivation to attempt to quit smoking, and this is interpreted as the smokers’ attempt to control their own risks and adverse consequences^6^. Moreover, perceived health status, attitude, and the extent of interest in health vary across individuals, and these factors may drive health-promoting behaviors^7^. An individual's attitude towards health and their level of interest influence the adoption of health-promoting behaviors, particularly quitting smoking^6,7^. Therefore, there is a need for targeted smoking cessation interventions that are strategically designed to align with the attitudes towards health among tobacco users.

However, these study findings have been obtained primarily from cigarette smokers and do not fully capture the evolving landscape of tobacco use or how the factors influencing quit attempts, and plans may differ based on the type of tobacco product or the user's status. In other words, there is a need to comprehensively understand and target the characteristics of diverse tobacco users. In this context, this study aims to compare attempts and plans to quit smoking according to user status among adult tobacco users in Korea, where diverse tobacco products have been commercialized. Additionally, the psychosocial and subjective health-related factors associated with quit attempts and plans will be examined, categorized by tobacco use status.

## METHODS

### Participants

The participants were recruited through a specialized survey firm. The firm sent an online survey link via email to their existing online panel members. The online survey was conducted from 21 November 2022, to 2 December 2022, and a total of 1288 adult tobacco users completed the survey.

A sample size estimation was performed using the G*Power software package (version 3.1.9.7; Kiel University)^8^. The input parameters included the following: statistical test = ANOVA: repeated measures, within factors; effect size f = 0.25; α error probability = 0.05; power (1 − β error probability) = 0.80; number of groups = 3, the calculated sample size (n = 400) satisfied the required condition (n = 269). The goal was to recruit 400 adult participants for each type of tobacco product, specifically cigarettes, e-cigarettes, and HTPs, and each user group was recruited proportionately by sex, age, and regional proportions of the Korean population.

### Measures

*Tobacco users*

Current cigarette users were defined as individuals who have consumed at least 100 cigarettes in their lifetime and currently smoke cigarettes daily or occasionally^9^. E-cigarettes refer to electronic devices typically comprising a cartomizer and a battery, and in this study, individuals who use e-cigarettes on a regular or occasional basis were defined as current e-cigarette users^9^. Current HTP users were defined as individuals who use HTP (products for inhalation of special sticks such as IQOS, GLO, and LIL after heating) daily or occasionally^9^.

*Self-reported health conditions, stress, health concerns, health behavior*

Self-reported health was assessed using a single question: ‘How would you rate your health?’. Response options were: 1=very bad, 2= bad, 3=moderate, 4=good, and 5=very good^10^. A higher score represents better self-reported health.

Self-reported stress was also assessed using a single question: ‘How much stress do you have in your day-to-day life?’. Response options were: 1=almost none, 2=little, 3=much, and 4=very much^10^. A higher score represents greater self-reported stress.

Health awareness was assessed using five statements: ‘Maintaining optimal health is very important for me’; ‘I will be able to remain healthy throughout the rest of my life if I enjoy a good diet, exercise, and engage in preventive behaviors’; ‘My health depends on how I take care of it’; ‘I actively try to prevent disease’; and ‘I do my best to maintain good health’. Response options were: 1=strongly disagree, 2=disagree, 3=neutral, 4=agree, and 5=strongly agree. The health concerns score was a sum of the responses to the five statements, and a higher score represents greater health concerns.

The health behavior score was calculated by summing the scores for each of the health behaviors performed routinely or in the past year (health checkups, low-fat diet/routine, fruit, vegetable, and grain intake, drinking adequate amounts of water, use of supplements (such as vitamins and minerals), regular exercise, no consumption or moderate consumption of alcohol, maintaining a healthy body weight). A higher number of items represents better health behaviors.

*Self-reported addiction*

The participants were asked to score their dependence on tobacco products on a scale from 0 to 100, where 0 is not dependent at all, and 100 is extremely dependent^11^. Thus, a higher score represents greater self-reported tobacco dependence. Next, the participants were asked how soon they use a tobacco product after they wake up in the morning^11^. Tobacco addiction is generally assessed by categorizing the responses into within 5 minutes, within 15 minutes, within 30 minutes, and within 60 minutes^12^. In our study, a longer time until tobacco product use represents lower addiction.

*Intention to quit using tobacco products*

Regardless of the current tobacco product used, all participants were asked whether they had stopped using their tobacco product for at least one day (≥24 hours) in the past year as an attempt to quit using it. A ‘yes’ response was considered to have attempted to quit smoking, and ‘no response was considered to have made no attempts to quit smoking.

*Plan to quit using tobacco products*

Likewise, all participants were asked whether they had plans to quit using tobacco products, regardless of which tobacco product they were currently using. The responses were categorized into: plans to quit within a month; plans to quit within six months; plans to quit someday, although not within six months; and no plans at all. For the analysis, ‘within one month’, ‘within six months’, and ‘someday’ were considered to have plans to quit smoking, and ‘no plans at all’ was considered to have no plans to quit.

*Covariates*

All covariates were measured using an online questionnaire. These included participants’ demographic characteristics: sex (male, female), age (20–29, 30–39, 40–49, 50–59, 60–69 years), region (metropolitan, non-metropolitan area), education level (high school or lower, college or higher), monthly income (<3, 3–4.9, 5–6.9, ≥7 million KRW), and occupation (manager, office worker, service and manual laborer, other).

### Data analysis

Data were analyzed using SAS version 9.4 (SAS Institute, Inc.; Cary, NC) software. The varying distributions of tobacco user groups according to general characteristics were analyzed by performing chi-squared tests. The differences in the mean scores of health concerns, health behavior, self-rated health, self-rated stress, self-rated addiction, and time to first tobacco use in the morning, according to the tobacco user groups were analyzed with one-way ANOVA using *post hoc* comparisons by Tukey HSD. The predictors of attempts and plans to quit using tobacco were identified using multiple linear regression and analyzed at a significance level set at p<0.05, controlling for demographic characteristics.

## RESULTS

### Participants’ demographic characteristics

Of 1288 tobacco users, there were 713 (55.4%) single users [cigarette users, 476 (66.8%); e-cigarette users, 93 (13.0%); HTP users, 144 (20.2%)], 364 (28.3%) dual users [cigarette + e-cigarette users 219 (60.2%); cigarette + HTP users 109 (29.9%); e-cigarette + HTP users 36 (9.9%)], and 211 (16.4%) triple users (Table 1). Although the percentage of men was higher in each tobacco user group, the difference was not significant (p=0.483). The predominant age group was 50–59 years among single users, 40–49 years among dual users, and 20–29 years among triple users (p<0.0001). While more single users lived in a non-metropolitan than in a metropolitan area, the opposite was true among dual users and triple users (p=0.0033). By education level, the percentage of users with a college degree or higher was higher among triple users compared with other user groups (p=0.0002). In terms of income, the most common monthly income was 3–4.9 million KRW among single and dual users but 5–6.9 million KRW among triple users (p=0.0003). The percentage of office workers was higher among dual users and triple users than single users (p<0.0001). Most users in each group lived with family as opposed to living alone, but this percentage was the highest among triple users (p=0.0465).

**Table 1:** Participants’ demographic characteristics, South Korea 2022 (N=1288)

*Characteristics*	*Single users (N=713)*		*Dual users (N=364)*		*Triple users (N=211)*		p*
	*n*	*%*	*n*	*%*	*n*	*%*	
**Sex**							0.483
Male	428	60.0	217	59.6	117	55.5	
Female	285	40.0	147	40.4	94	44.5	
**Age** (years)							<0.0001
20–29	150	21.0	77	21.2	70	33.2	
30–39	119	16.7	76	20.9	50	23.7	
40–49	147	20.6	94	25.8	45	21.3	
50–59	175	24.5	67	18.4	25	11.8	
60–69	122	17.1	50	13.7	21	10.0	
**Region**							0.0033
Metropolitan	327	45.9	203	55.8	115	54.5	
Non-metropolitan	386	54.1	161	44.2	96	45.5	
**Education level**							0.0002
High school or lower	166	23.3	55	15.1	27	12.8	
College or higher	547	76.7	309	84.9	184	87.2	
**Monthly income** (million KRW)							0.0003
<3	208	29.2	67	18.4	48	22.7	
3–4.9	244	34.2	130	35.7	57	27.0	
5–6.9	153	21.5	99	27.2	58	27.5	
≥7	108	15.1	68	18.7	48	22.7	
**Occupation**							<0.0001
Manager	78	10.9	41	11.3	20	9.5	
Office worker	306	42.9	200	54.9	129	61.1	
Service/labor worker	192	26.9	80	22.0	35	16.6	
Other	137	19.2	43	11.8	27	12.8	
**Living arrangement**							0.0465
Living with family	408	57.2	229	62.9	138	65.4	
Living alone	305	42.8	135	37.1	73	34.6	
**Quit attempt**							0.0004
Yes	371	52.0	199	54.7	142	67.3	
No	342	48.0	165	45.3	69	32.7	
**Quit plan**							0.0349
Yes	575	80.6	313	86.0	182	86.3	
No	138	19.4	51	14.0	29	13.7	

* Chi-squared analysis. KRW: 1 million Korean Won about US$770.

The percentage of users who have attempted to quit was higher than those who have never attempted to quit in all three user groups, but this percentage was the highest in the triple user group (p=0.0004). Moreover, there were more users with a plan to quit than those without a plan to quit in all three user groups, but this percentage was higher among dual and triple users than single users (p=0.0349).

### Comparison of self-rated health and self-rated tobacco addiction among tobacco user groups

**Figure 1:**
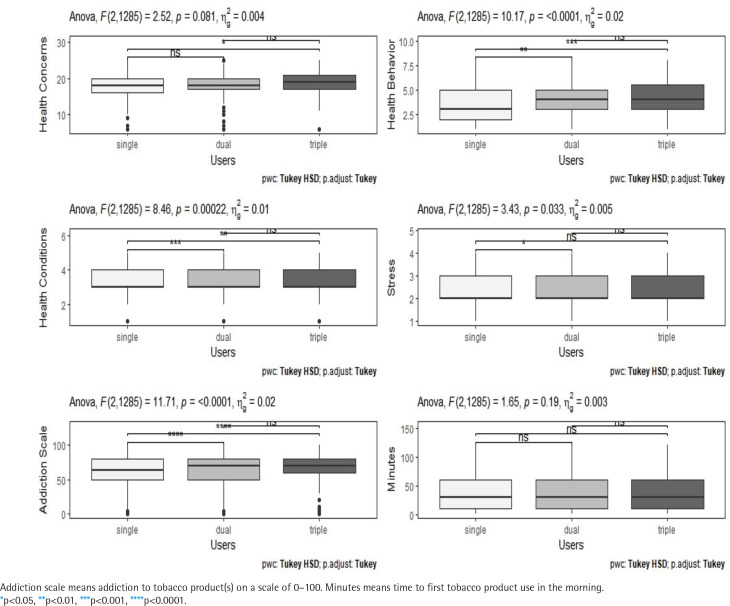
Comparison of self-rated health and self-rated tobacco addiction among tobacco user groups, South Korea 2022 (N=1288)

Figure 1 presents the comparison of self-rated health across the tobacco user groups. Although statistically insignificant, the health concern score was the highest among triple users, followed by dual users and single users (p=0.0808). Likely, the health behavior score was the highest among triple users, followed by dual users and single users (p<0.0001). The self-rated health condition score was lower among single users than dual users and triple users (p=0.0002), and the self-rated stress score was the highest among triple users and lowest among single users (p=0.0326).

Regarding self-rated tobacco addiction, the self-rated addiction score was the highest among triple users, followed by dual and single users (p<0.0001). Although not statistically significant, the time to first tobacco use in the morning was the longest among single users, followed by dual users and triple users (p=0.1922).

### Predictors of quit attempt and quit plans

Table 2 shows the results of multiple linear regression analyzing the association between self-rated health, self-rated addiction and quit attempts, controlling for demographic characteristics. Among single users, health behaviors (β=0.135, p=0.002) or time to first tobacco use in the morning (β=0.139, p<0.001) were positively related to the quit attempts, but self-rated addiction scales (β= -0.143, p<0.001) were negatively related to the quit attempts. In the dual-user group, quit attempts were significantly related to health behaviors (β=0.183, p=0.002) but were negatively related to stress (β= -0.139, p=0.01) or addiction scale (β= -0.119, p=0.03). Among triple users, none of the factors predicted quit attempts. Table 2 shows the results of multiple linear regressions analyzing the association between self-rated health, self-rated addiction, and quit attempts, controlling for demographic characteristics. Among single users, health behaviors (β=0.135, p=0.002) or time to first tobacco use in the morning (β=0.139, p<0.001) were positively related to the quit attempts, but self-rated addiction scales (β= -0.143, p<0.001) were negatively related to the quit attempts. In the dual-user group, quit attempts were significantly related to health behaviors (β=0.183, p=0.002) but were negatively related to stress (β= -0.139, p=0.01) or the addiction scale (β= -0.119, p=0.03). Among triple users, none of the factors predicted quit attempts. Table 3 presents the results of multiple linear regression analyzing the association between self-rated health, self-rated addiction, and quitting plans. Health behavior predicted quitting plans in all tobacco user groups. Among single and dual users, quitting plans were positively related to the time to first tobacco use in the morning (β=0.166, p<0.001). In single users, the self-rated addiction scale was negatively related to the quit plan (β= -0.131, p=0.001).

**Table 2:** Multiple linear regression results* of the attempt to quit the use of tobacco products, South Korea 2022

*Variable*	*Single users*					*Dual users*					*Triple users*				
	*Unstandardized coefficients*		*Standardized coefficients*	*t*	*p*	*Unstandardized coefficients*		*Standardized coefficients*	*t*	*p*	*Unstandardized coefficients*		*Standardized coefficients*	*t*	*p*
	*B*	*SE*	*β*			*B*	*SE*	*β*			*B*	*SE*	*β*		
Health concerns	0.000	0.005	0.004	0.090	0.930	0.003	0.006	0.024	0.420	0.672	0.003	0.009	0.026	0.320	0.748
Health behavior	0.027	0.009	0.135	3.160	0.002	0.037	0.012	0.183	3.120	0.002	0.009	0.013	0.052	0.660	0.512
Health conditions	-0.031	0.021	-0.058	-1.460	0.143	0.031	0.026	0.065	1.180	0.238	-0.015	0.039	-0.030	-0.370	0.710
Stress	-0.005	0.024	-0.008	-0.200	0.843	-0.075	0.029	-0.139	-2.590	0.010	-0.038	0.036	-0.080	-1.070	0.284
Addiction scale	-0.002	0.001	-0.143	-3.660	0.000	-0.002	0.001	-0.119	-2.180	0.030	0.000	0.001	-0.003	-0.040	0.970
Minutes	0.001	0.000	0.139	3.640	0.000	0.000	0.001	0.014	0.260	0.797	-0.001	0.001	-0.065	-0.860	0.389

* The results represent the full model containing all covariates; sex, age, region, education level, monthly income, occupation, and living arrangement.

**Table 3:** Multiple linear regression results* of the plan to quit the use of tobacco products, South Korea 2022

*Variable*	*Single users*					*Dual users*					*Triple users*				
	*Unstandardized coefficients*		*Standardized coefficients*	*t*	*p*	*Unstandardized coefficients*		*Standardized coefficients*	*t*	*p*	*Unstandardized coefficients*		*Standardized coefficients*	*t*	*p*
	*B*	*SE*	*β*			*B*	*SE*	*β*			*B*	*SE*	*β*		
Health concerns	0.001	0.006	0.008	0.200	0.842	-0.001	0.009	-0.007	-0.130	0.897	0.017	0.012	0.111	1.430	0.155
Health behavior	0.054	0.011	0.211	5.090	<0.0001	0.044	0.017	0.152	2.610	0.010	0.043	0.017	0.195	2.520	0.013
Health conditions	-0.009	0.026	-0.013	-0.350	0.729	0.028	0.038	0.040	0.740	0.462	0.010	0.052	0.015	0.200	0.843
Stress	0.024	0.029	0.030	0.810	0.416	-0.015	0.041	-0.019	-0.350	0.724	0.034	0.047	0.053	0.730	0.468
Addiction scale	-0.003	0.001	-0.131	-3.460	0.001	-0.002	0.001	-0.100	-1.830	0.068	-0.001	0.001	-0.048	-0.680	0.497
Minutes	0.002	0.000	0.166	4.460	<0.0001	0.003	0.001	0.174	3.220	0.001	0.000	0.001	0.017	0.240	0.812

* The results represent the full model containing all covariates; sex, age, region, education level, monthly income, occupation, and living arrangement.

Table 3 presents the results of multiple linear regression analyzing the association between self-rated health, self-rated addiction and quitting plans. Health behavior predicted quitting plans in all tobacco user groups. Among single and dual users, quitting plans were positively related to the time to first tobacco use in the morning (β=0.166, p<0.001). In single users, the self-rated addiction scale was negatively related to the quit plan (β= -0.131, p=0.001).

## DISCUSSION

In this study, we observed significant differences in past quit attempts and future quitting plans according to the number of tobacco products currently used. Comparatively, a significantly higher proportion of individuals who are dual or triple users have made attempts to quit using tobacco and have future plans to quit, in contrast to those who are single users. This finding suggests that tobacco users in Korea have the ability to use multiple tobacco products simultaneously as a means of smoking cessation. This is supported by numerous studies indicating that many e-cigarette users utilize e-cigarettes as a means to quit smoking^3,13,14^. Reports indicate a significant increase in the percentage of individuals attempting to quit cigarette smoking as the use of e-cigarettes increases.

However, the quit attempts or plans described here may only refer to quitting cigarette smoking and not necessarily other e-cigarette products. The World Health Organization (WHO) has advised caution regarding the use of e-cigarettes, citing a lack of evidence regarding their safety and effectiveness in smoking cessation^15^. Nevertheless, many e-cigarette users perceive these devices to be safe^16,17^. Therefore, cessation approaches for dual and triple users need to be designed to lead to the cessation of all tobacco product use, not just cigarettes, by concurrently using other tobacco products.

Additionally, we observed that participants' health behaviors significantly differ depending on the tobacco products they use. Compared to single users, dual users and triple users demonstrated a higher rate of health behaviors, and positive health behaviors were identified as a potent predictor of tobacco quit attempts or plans. In particular, health behavior was the only predictor of quitting plans among triple users. This suggests that the high rate of quit attempts or plans among dual and triple users was part of their health practices based on their health awareness. Moreover, these results can indicate that multiple tobacco product users engage in several health behaviors to offset the health problems that may potentially arise from the use of several tobacco products, and, as a result, they rate their general health highly.

It is noteworthy that dual and triple users rated their health more highly than single users. In general, self-rated health among smokers declines with the increasing length of smoking, and the self-rated health of past smokers improves to levels comparable with that of non-smokers over time^18^. However, our results showed differences in self-rated health among different tobacco user groups, although not compared with non-smokers, suggesting that multiple tobacco product users are more confident in their health than single users. These findings may suggest that individuals who use multiple tobacco products may not hesitate to use them because they perceive themselves to be healthy. Conversely, however, they may view the use of multiple tobacco products as acceptable because they engage in various health behaviors and practices. Therefore, this may be attributable to the fact that the determinants of self-rated health could be their expectations for their health and independent functioning, as opposed to their pre-existing chronic conditions.

We also found significant differences in self-rated addiction to tobacco products based on the number of products used. Specifically, dual and triple users reported higher self-reported addiction scores and had a shorter time to first tobacco use after waking up in the morning compared with single users. These findings are consistent with prior research, which also suggests that poly users have higher levels of addiction compared with single users^3^. There are several explanations for the higher levels of addiction among poly users. One possibility is that cigarette users may have used e-cigarettes as a secondary means to quit smoking, as discussed earlier. Another possibility is that cigarette users use multiple tobacco products because they want to use them more freely, comfortably, and without restrictions. In this regard, studies suggest that cigarette users often use e-cigarettes concurrently because they can be used indoors. These findings highlight the need for more effective intervention strategies to help poly users quit smoking, as they may have significant levels of addiction due to unsuccessful attempts at quitting tobacco products and the concurrent use of multiple products.

In particular, even single users with higher levels of addiction were less likely to attempt or plan to quit smoking, calling for effective measures to nudge them from the pre-contemplation stage to the contemplation stage in terms of quitting smoking^19^. In this regard, studies have shown that intentions to quit tobacco use increase as smokers develop an increased awareness of the benefits of quitting or the severity of the harms caused by tobacco use^20^. Therefore, effectively communicating that simply using one or more tobacco products may pose significant health risks, is important. Tobacco dependence often requires multiple attempts to quit, and quitting can be a challenging process with repeated cycles of success and failure^21^. However, establishing a plan to prepare for quitting is an important step to quitting smoking.

Another notable finding is that users who are normally under high stress do not consider quitting the use of tobacco products. In general, tobacco users point to stress relief as their primary reason for using tobacco products, but in reality, stress is known to not only increase smoking but also decrease self-efficacy for smoking cessation and relapse to smoking after quitting^22^. These results suggest that stress management is an important element in smoking cessation. Therefore, smoking cessation programs should pay close attention to the role of stress during cessation attempts to improve success rates. Research suggests that successful smoking cessation is psychologically linked to reduced depression, anxiety, and stress, as well as an improved quality of life compared with their previous lives as smokers. In other words, addressing concerns about life without tobacco may be an effective strategy to motivate individuals to attempt to quit smoking^23,24^.

One academic implication of this study pertains to presenting effective intervention measures for smoking cessation by identifying the predictors of quit attempts and quit plans according to the number of tobacco products used.

### Limitations

This study has some limitations. First, our study data were collected from 1288 adults recruited online, so the sample is not representative of the Korean population, and therefore, the findings cannot be generalized to the entire population. However, to address this issue, we attempted to enroll participants considering sex, age, and regional distributions in Korea. Second, we used a self-report questionnaire, so the accuracy of self-rated health and self-rated addiction is not guaranteed. Particularly since there is no established instrument to measure the level of addiction based on the number of tobacco products currently used, we modified an instrument developed to measure nicotine dependence among cigarette and e-cigarette users, which leaves the risk of measurement bias. Third, tobacco users were categorized as single, dual, and triple users based on the number of tobacco products they use. However, individual variations within each group, such as differences in the amount of consumption, frequency of use, length of use, and specific products used, were not considered. Therefore, there may be substantial variations across individuals, even within the same user group. Fourth, the survey was conducted in November 2022, when mandatory use of face masks indoors and mandatory quarantine of individuals with COVID-19 were still in place, so the COVID-19 pandemic may have influenced the results. This is because COVID-19 has not only had tremendous social repercussions but has reportedly also had profound impacts on tobacco use patterns among tobacco users. Therefore, longitudinal studies should be conducted to address these limitations.

## CONCLUSIONS

This study’s findings indicate that the likelihood of quitting tobacco products or having plans to quit varies based on the number of tobacco products currently used. There are also significant differences in subjective health and levels of tobacco product addiction across users. Notably, quit attempts and plans increased with the increasing practice of health behaviors, indicating that users may perceive using various types of tobacco products as part of healthy practices, such as quitting smoking. Thus, imparting accurate information about the health effects of tobacco products is important to motivate users to attempt and plan to quit, and continue abstinence. Moreover, based on the results that quit attempts or quit plans decreased with increasing levels of self-rated addiction, effective interventions, such as stress management to promote successful quitting, should be developed for individuals with severe addiction.

## Data Availability

The data supporting this research cannot be made available for privacy or other reasons.
